# Rotavirus symptomatic infection among unvaccinated and vaccinated children in Valencia, Spain

**DOI:** 10.1186/s12879-019-4550-x

**Published:** 2019-11-27

**Authors:** Raúl Pérez-Ortín, Cristina Santiso-Bellón, Susana Vila-Vicent, Noelia Carmona-Vicente, Jesús Rodríguez-Díaz, Javier Buesa

**Affiliations:** Department of Microbiology, School of Medicine, University of Valencia and Microbiology Service, Hospital Clínico Universitario and Instituto de Investigación INCLIVA, Avda. Blasco Ibañez, 17, 46010 Valencia, Spain

**Keywords:** Gastroenteritis, Rotavirus, Vaccine, Genotype

## Abstract

**Background:**

Human group A rotavirus is the leading cause of severe acute gastroenteritis in young children worldwide. Immunization programs have reduced the disease burden in many countries. Vaccination coverage in the Autonomous Region of Valencia, Spain, is around 40%, as the rotavirus vaccine is not funded by the National Health System. Despite this low-medium vaccine coverage, rotavirus vaccination has substantially reduced hospitalizations due to rotavirus infection and hospital-related costs. However, there are very few studies evaluating symptomatic rotavirus infections not requiring hospitalization in vaccinated children. The objective of this study was to investigate symptomatic rotavirus infections among vaccinated children in the health area served by the Hospital Clínico Universitario of Valencia, Spain, from 2013 to 2015.

**Methods:**

A total of 133 children younger than 5 years of age with rotavirus infection were studied. Demographic and epidemiological data were collected and informed consent from their caretakers obtained. Rotavirus infection was detected by immunological methods and G/P rotavirus genotypes were determined by RT-PCR, following standard procedures from the EuroRotaNet network.

**Results:**

Forty infants (30.1%; 95% CI: 22.3–37.9) out of 133 were diagnosed with symptomatic rotavirus infection despite having been previously vaccinated, either with RotaTeq (85%) or with Rotarix (15%). Children fully vaccinated against rotavirus (24.8%), partially vaccinated (5.3%) and unvaccinated (69.9%) were found. The infecting genotypes showed high G-type diversity, although no significant differences were found between the G/P genotypes infecting vaccinated and unvaccinated children during the same time period. G9P[8], G12P[8] and G1P[8] were the most prevalent genotypes. Severity of gastroenteritis symptoms required 28 (66.6%) vaccinated and 67 (73.6%) unvaccinated children to be attended at the Emergency Room.

**Conclusion:**

Rotavirus vaccine efficacy in reducing the incidence of severe rotavirus infection has been well documented, but symptomatic rotavirus infection can sometimes occur in vaccinees.

## Background

Group A rotaviruses are the leading cause of acute gastroenteritis in infants and young children worldwide. At 5 years of age almost every child has suffered at least one rotavirus infection [1]. Moreover, the same child is likely to become infected several times during the first years of life, although the symptomatology of the second infection is usually milder and the third is typically asymptomatic [2]. In adults, rotavirus infection is usually asymptomatic or mild, although it can be severe in immunocompromised individuals or the elderly [3]. Both infected children and adults shed rotaviruses in their stools, causing the virus to spread easily within the community [4]. Rotavirus is also an major cause of nosocomial diarrhoea [5].

In the years prior to the introduction of rotavirus vaccines, this virus was responsible for 440,000 deaths per year [6]. According to the most recent study of the global burden of disease, rotavirus infection was responsible in 2016 for an estimated 128,500 deaths (95% uncertainty interval [UI],104,500 - 155,600) among children younger than 5 years; thus, 28.8% (95% UI, 25.0–32.6%) of the deaths from diarrhea in this age group were attributable to rotavirus [7]. In developing countries almost all these deaths are due to dehydration and related to poor health care [1]. In developed countries mortality is very low, but rotavirus infection is the main cause of child hospitalization due to acute gastroenteritis (2 million hospitalizations each year) and is responsible for 25 million outpatient consultations each year worldwide [8]. In Spain, mortality due to rotavirus is almost non-existent; however, rotavirus is responsible for 14 to 30% of all cases of acute gastroenteritis and of these, a quarter require hospitalization [9]. Rotavirus acute gastroenteritis costs the Spanish National Health System around 28 million euros per year [10].

Since 2006 two live attenuated oral rotavirus vaccines have been marketed worldwide: the monovalent Rotarix® (GSK Biologicals, Rixensart, Belgium), an attenuated human G1P[8] vaccine, and the pentavalent bovine-human mono-reassortant RotaTeq® vaccine (Merck and Co, PA, USA), which contains human rotavirus VP4 (P[8]) and VP7 (G1-G4) genes in a bovine rotavirus genetic background [11]. The two vaccines have been highly effective in industrialized countries [12, 13], but studies carried out in sub-Saharan African countries showed far lower efficacy in these geographical areas [14, 15]. WHO recommends the inclusion of rotavirus vaccine in the National Childhood Immunization Program in countries with high incidence of rotavirus infection [16]. Their implementation in many countries has demonstrated a reduction in the disease burden of rotavirus diarrhoea and related death in several geographic regions worldwide [17–20] including sub-Saharan African countries [21]. Rotavirus vaccines are also recommended by the Spanish Paediatric Association since 2008 [22], but they are not funded by the National Health System. In Spain, parents pay for the vaccine, and vaccination coverage has reached around 40% in the Autonomous Region of Valencia [23]. Despite this low-medium vaccine coverage, the introduction of rotavirus vaccines has had a specific coverage-related impact on hospitalizations due to acute gastroenteritis in children under 5 years of age, and their use has substantially reduced hospital-related costs [24].

After rotavirus vaccine implementation, the prevalence of rotavirus diarrhoea in children has changed [25–27], but there are few studies evaluating symptomatic infections not requiring hospitalization in vaccinated children [28, 29]. The purpose of this study was to investigate the presence of symptomatic rotavirus infections among vaccinated infants and children in the city of Valencia, Spain between January 2013 and December 2015.

## Methods

### Study population and sample collection

The study was carried out with paediatric patients from the health area of the Hospital Clínico Universitario of Valencia. The total population attended by this hospital is 345,498, of whom 20,091 (5.82%) are children under 5 years of age. In the Autonomous Region of Valencia rotavirus vaccination is not included in the Childhood Vaccination Programme, and its use is restricted to private healthcare or to the initiative of parents and guardians.

The study protocol and the informed consent given by caretakers of the children included in the study was approved by the Clinical Research Ethics Committee of the Hospital Clínico Universitario of Valencia (Approval No. F-CE-GEva-15) and by the Juvenile Prosecutor’s Office of Valencia (ref. 33/15).

During the first study phase data were collected between January 2013 and December 2015 from a total of 217 patients with acute gastroenteritis due to rotavirus, who were attended either at the Primary Care Paediatrics Clinics served by the Hospital Clínico Universitario of Valencia or at the Paediatrics Emergency Room of the same hospital. Once the data had been collected, the children’s parents or caregivers were requested permission to enrol them in the study, which was obtained for 133 children. Of these, 71 patients (53.4%) were inhabitants of the metropolitan area of Valencia and 62 patients (46.6%) were from rural areas served by the Hospital Clínico Universitario of Valencia.

In order to make a comparative analysis we included a control group of 50 healthy children, 24 boys (48%; CI95%: 33.7–62.6) and 26 girls (52%; CI95%: 37.4–66.3), with similar demographic characteristics to the patient group. Age at time of analysis ranged from 1 month to 5 years, with an average of 2 years and 10 months; 31 (62%) were under 3 years of age. Concerning rotavirus vaccination, 28 children (56%; CI95%: 41.3–70) had been vaccinated.

### Detection of rotavirus in stool samples

Detection of rotavirus antigen in stool samples was performed by an immunochromatographic test (Rotavirus-Adenovirus, Certest Biotec, Spain) (in 173 samples, 79.7%) or by ELISA (Premier™ Rotaclone, Meridian Bioscience, Cincinnati, OH, US) (in 44 samples, 20.3%) following the manufacturer’s instructions. The sensitivity and specificity of the Premier™ Rotaclone® assay have been reported to be 76.8 and 100% compared to an RT-PCR assay, respectivey [30], whereas the sensitivity and specificity of the CerTest Rotavirus+Adenovirus ICG test for detection of rotavirus are 99 and 98%, respectively, compared with a commercial ELISA test (Ridascreen®Rotavirus, R-Biopharm, Germany) and according to the manufacturer.

### Diagnosis of other enteric pathogens

To investigate possible concomitant enteropathogenic bacteria, stool samples were plated onto bacterial culture media, including selenite broth, MacConkey agar, Salmonella Shigella agar, cefsulodin-irgasan-novobiocin agar, charcoal cefoperazone deoxycholate agar (CCDA) and Columbia agar with 5% sheep blood. All culture media were from BD Diagnostics (Heidelberg, Germany). All the specimens were processed immediately upon arrival at the laboratory. Culture media were incubated at 37 °C for 24 h, except CCDA plates, which were incubated at 42 °C under microaerophilic conditions for 48 h. Colonies considered as presumptive enteric pathogens were identified with matrix-assisted laser desorption/ionization time of flight mass spectrometry (MALDI-TOF MS Biotyper 3.1, Bruker, MA, US). Other enteric viruses like astroviruses were investigated using ProSpecT Astrovirus Microplate EIA Assay from Oxoid (Thermo Fisher Scientific, MA, US) according to the manufacturer’s instructions, and noroviruses by RT-PCR as previously described [31].

### Characterization of rotavirus G and P genotypes

Rotavirus-positive samples were confirmed by RT-PCR and the infecting rotavirus genotypes were identified. Faecal suspensions at 10–20% (w/v) were prepared in phosphate buffered saline (PBS) pH 7.2. Viral RNA was extracted by the guanidine isothiocyanate and silica matrix method (Boom method) [32] or by extraction with Trizol® (Invitrogen) [33]. The extracted RNA was kept at − 80 °C until processed by reverse transcription and PCR.

The reverse transcription reaction was performed with random primers using SuperScript® III reverse transcriptase (Life Technologies, Thermo Fisher Scientific, MA, US). The encoding genes of rotavirus proteins VP7 and VP4 were amplified in two different PCR reactions. Subsequently, ‘multiplex’ PCR reactions were used to identify the genotype G (VP7) and P (VP4) of each rotavirus strain, following previously procedures described [34] and standardized by the European Rotavirus Surveillance Network, “EuroRotaNet” (www.eurorotanet.com).

### Statistical analysis

Data were analysed using the R Core Team 2015 software (version 3.2.2). The estimated frequency was calculated as a percentage with a 95% confidence interval for each variable. To compare them, Chi-square test was used, except in cases in when the frequency was less than 5, in which Fisher’s exact test was applied. Significant differences were assumed for *p* values < 0.05. The results obtained for each of the variables were compared using the median-unbiased estimation method. For this purpose, the first category of each variable was taken as a reference and compared to the remaining categories analysed for the same variable [35].

## Results

### Demographic and clinical features of the study population

**Fig. 1:**
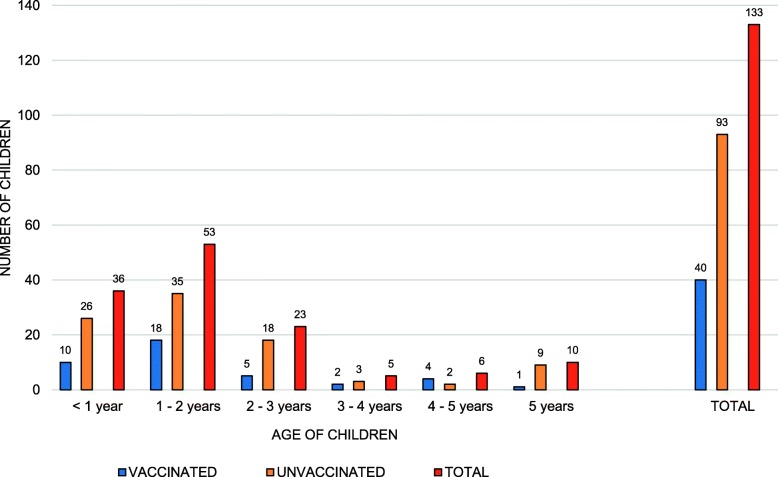
Distribution by age and rotavirus vaccination status of children. Highest vaccine coverage occurs in children under 3 years of age. The vaccinated group includes full (33) and partially (7) vaccinated children

Patient ages ranged from 13 days to 5 years, average 22 months. Most children (112, 84.2%) were under 3 years of age, 62 were female (46.6%; CI95%: 37.9–55.5) and 71 were male (53.4%; CI95%: 44.5–62.1). Regarding rotavirus vaccination status, 93 patients (69.92%; CI95%: 62.1–77.7) had not received any vaccine dose at the time of infection (Fig. 1). Twenty-eight (66.6%) vaccinated children and 67 (73.6%) unvaccinated children were attended at the Paediatric Emergency Room due to severity of symptoms.

### Infecting rotavirus genotypes

**Fig. 2:**
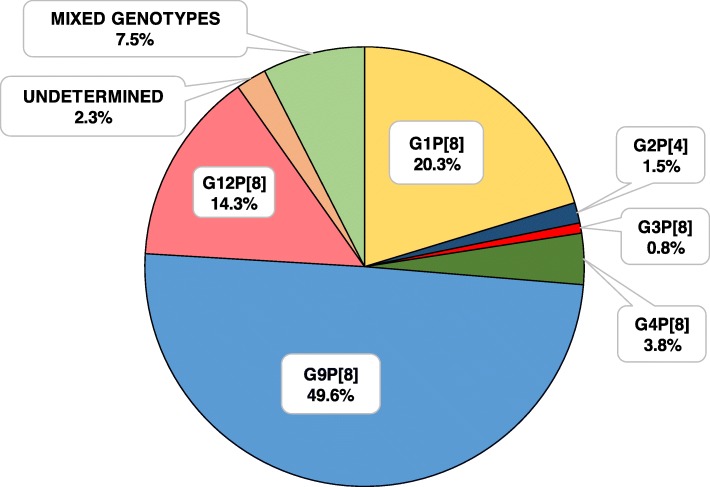
Distribution of rotavirus G/P genotypes. Ninety-eight percent of the rotavirus strains detected corresponded to the P[8] genotype, predominantly the G9[P8] genotype

**Fig. 3:**
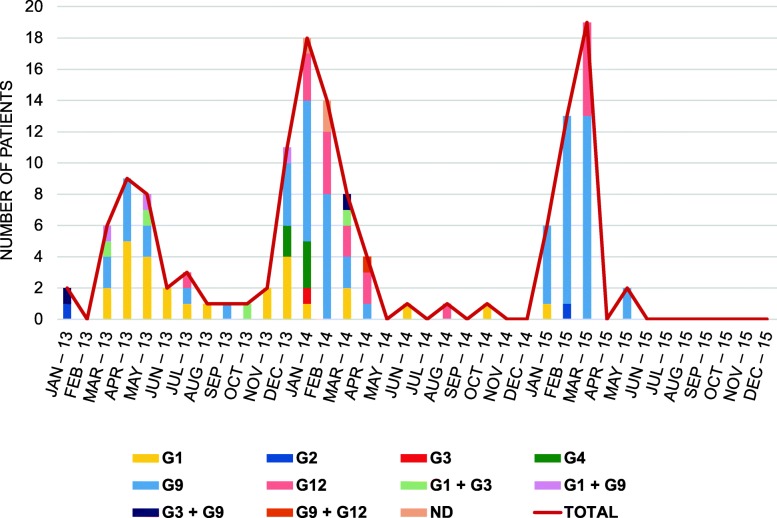
Temporary distribution of G genotypes of infecting rotavirus. Significant variations were observed in the incidence of rotavirus infection as well as in the relative frequency of the G genotypes detected during the study

Most patients (90.2%) were infected by a single rotavirus genotype and in those with mixed infections (7.5%) two different genotypes were detected (Fig. 2). The most frequently found P genotype was P[8] (97.7%; CI95%: 93.5–99.5) and only two patients were infected by a P[4] genotype (1.5%; CI95%: 0.2–5.3). Regarding G genotype, G1, G2, G3, G4, G9 and G12 were detected. Temporary distribution of rotavirus G genotypes along the study period is shown in Fig. 3. The predominant G/P genotype was rotavirus G9P[8] (49.6%; CI95%: 40.8–58.4), followed by G1P[8] (20.3%; CI95%: 13.8–28.1) and G12P[8] (14.3%; CI95%: 8.8–21.4). Peak infection periods occurred during the coldest months of the year (November to April), with G9P[8] genotype being the most prevalent during these months in the 2013–14 and 2014–15 seasons. In 2013, in addition to the winter peak, there was an increase in rotavirus infections from March to July due to the G1P[8] genotype. The number of G1P[8] genotype infections remained stable for most of the study period, with minimal oscillations during seasons. Although rotavirus infections were detected from June to December 2015, the caretakers of those patients did not sign informed consent to participate in the study.

### Rotavirus G/P genotypes detected in vaccinated children

Forty infants (30.1%; CI95%: 22.3–37.9) out of the 133 diagnosed with rotavirus infection had been previously vaccinated. Of these, 33 had been fully vaccinated, whereas 7 children received an incomplete number of vaccine doses. The infecting genotypes showed high G genotype diversity (Table 1), although no significant differences were found between the G/P genotypes infecting vaccinated and unvaccinated children (*p* = 0.706) (Table 2). Twenty-eight (66.6%) vaccinated children and 67 (73.6%) unvaccinated children were attended at the Paediatric Emergency Room due to severity of symptoms.

**Table 1:** Distribution of rotavirus G/P genotypes in 40 vaccinated children who developed rotavirus diarrhoea

Rotavirus Genotype	Number of children (*n* = 40) (%)	
G1P[8]	6	(15)
G4P[8]	2	(5)
G9P[8]	20	(50)
G12P[8]	8	(20)
G1P[8] + G9P[8]	2	(5)
G3P[8] + G9P[8]	2	(5)

**Table 2:** Rotavirus genotypes in vaccinated and unvaccinated children

Rotavirus Genotype	Vaccinated ^a^ (*n* = 44) (%)		Unvaccinated ^b^ (*n* = 98) (%)		Odds ratio (CI 95%)		*p* value
G1P[8]	8^c^	(18.2)	26^f^	(26.6)	–		–
G2P[4]	0	–	2	(2)	1.60	(0.07–36.80)	0.768
G3P[8]	2^d^	(5)	5^g^	(5.1)	0.77	(0.12–4.75)	0.777
G4P[8]	2	(5)	3	(3)	0.46	(0.06–3.27)	0.439
G9P[8]	24^e^	(54.6)	47^h^	(48)	0.60	(0.24–1.53)	0.287
G12P[8]	8	(18.2)	12^i^	(12.3)	0.46	(0.14–1.52)	0.205
ND	0	–	3	(3)	2.24	(0.10–48)	0.605

*ND* Not determined

^a^7 patients were partially vaccinated: one patient vaccinated with 1 dose of Rotarix© and 6 patients vaccinated with 1 dose of RotaTeq©

^b^In 3 patients it was not possible to determine the infecting genotype

^c^2 in mixed infection / ^d^ 2 in mixed infection / ^e^ 4 in mixed infection / ^f^ 5 in mixed infection / ^g^ 5 in mixed infection / ^h^ 3 in mixed infection / ^i^ 1 in mixed infection

### Detection of other enteric pathogens

None of other investigated enteropathogenic bacteria or viruses (enteric adenoviruses, astroviruses and noroviruses) were detected in these patients by conventional methods.

### Relationship between rotavirus infection and vaccination status

In our study, 30.1% (CI95%: 22.3–37.9) of rotavirus-infected patients had been previously vaccinated with one of the two vaccines marketed in Spain. The proportion of vaccinated children in the group of patients with rotavirus infection was lower than in the control group of uninfected children (OR: 2.96; CI95%: 1.51–5.78; *p* = 0.003) (Table 3). This is an interesting finding and supports the need for vaccination.

**Table 3:** Comparison of the ratio of vaccinated and unvaccinated children among rotavirus-infected patients and healthy controls. The data analysis shows a greater tendency of unvaccinated individuals to contract rotavirus infection. The type of vaccine, monovalent or pentavalent, was not taken into account

Vaccination status	Patients		Controls		Odds ratio		*p* value
	(*n* = 133) (%)		(*n* = 50) (%)		(CI 95%)		
Unvaccinated	93	(69.92)	22	(44.00)	–		–
Vaccinated	40	(30.08)	28	(56.00)	2.96	(1.51–5.78)	0.003

Table 4 shows the ages of the vaccinated children when they suffered rotavirus diarrhoea, the type of vaccine administered (monovalent Rotarix or pentavalent RotaTeq), the ages in weeks when doses were given, the time frame between last vaccine dose and infection, and the infecting rotavirus genotype. Four children were infected with rotavirus G9P[8] less than 2 months after the first vaccine dose. Because this genotype is not present in the composition of the vaccines faecal shedding of vaccine strains can be excluded. Another child was infected with rotavirus G9P[8] 5 days after having received the last dose.

**Table 4:** Features of the rotavirus infections in vaccinated children (age at time of infection, type of vaccine and doses given, time frame between last vaccine dose and infection, and infecting viral genotypes)

No.	Age at rotavirus infection		Vaccine given	First dose (age in weeks)	Second dose (age in weeks)	Third dose (age in weeks)	Time frame between last vaccine dose and infection		Infecting genotype
	Years	Months					Years	Months	
1	1	9	Rotarix®	8	16	NA	1	5	G1P[8]
2	4	9	RotaTeq®	8	Not given	Not given	4	7	G9P[8]
3	0	4	RotaTeq®	8	Not given	Not given	0	2	G9P[8]
4	3	2	RotaTeq®	8	16	24	2	8	G9P[8]
5	1	8	RotaTeq®	8	17	25	1	1	G9P[8]
6	1	9	RotaTeq®	9	15	24	1	3	G9P[8]
7	4	3	RotaTeq®	8	16	23	3	9	G9P[8]
8	0	4	RotaTeq®	7	Not given	Not given	0	1	G9P[8]
9	1	10	RotaTeq®	8	16	24	1	4	G9P[8]
10	1	6	RotaTeq®	8	17	23	1	0	G12P[8]
11	2	10	RotaTeq®	9	17	25	2	4	G9P[8]
12	0	10	RotaTeq®	8	16	24	0	4	G12P[8]
13	0	8	RotaTeq®	9	Not given	Not given	0	6	G12P[8]
14	2	0	RotaTeq®	8	17	25	1	5	G9P[8]
15	1	6	RotaTeq®	10	16	24	1	0	G4P[8]
16	0	11	RotaTeq®	8	16	24	0	5	G12P[8]
17	1	7	RotaTeq®	8	16	24	1	1	G9P[8]
18	1	8	RotaTeq®	12	18	24	1	2	G9P[8]
19	1	7	RotaTeq®	8	16	24	1	1	G9P[8]
20	0	6	RotaTeq®	7	20	27	0	7	G1P[8]
21	1	3	RotaTeq®	8	16	25	0	9	G9P[8]
22	2	7	RotaTeq®	8	17	24	2	1	G12P[8]
23	4	4	Rotarix®	9	17	NA	3	11	G12P[8]
24	0	7	RotaTeq®	9	Not given	Not given	0	5	G9P[8]
25	4	11	Rotarix®	10	17	NA	4	6	G9P[8]
26	1	5	RotaTeq®	9	17	25	0	11	G3 + G9P[8]
27	1	2	RotaTeq®	8	16	25	0	8	G12P[8]
28	3	1	RotaTeq®	8	16	24	2	6	G12P[8]
29	2	7	RotaTeq®	9	16	24	2	1	G9P[8]
30	0	9	RotaTeq®	10	17	25	0	3	G3 + G9P[8]
31	1	1	Rotarix®	8	16	NA	0	10	G1 + G9P[8]
32	1	8	RotaTeq®	9	17	25	1	2	G1P[8]
33	1	8	RotaTeq®	8	16	23	1	3	G9P[8]
34	1	7	RotaTeq®	8	16	24	1	1	G1P[8]
35	5	10	Rotarix®	12	Not given	NA	5	7	G1P[8]
36	1	4	RotaTeq®	7	16	23	0	11	G1 + G9P[8]
37	0	3	RotaTeq®	8	Not given	Not given	0	1	G9P[8]
38	2	8	RotaTeq®	9	16	24	2	2	G1P[8]
39	1	1	Rotarix®	12	23	NA	0	7	G4P[8]
40	0	6	RotaTeq®	9	17	25	0	0	G9P[8]

*NA* Not applicable

Not given doses denote partially vaccinated children

## Discussion

Throughout the study period, the incidence of rotavirus infections fluctuated significantly both in number of cases and prevalence of different infecting G genotypes, as was expected in a temperate climate region [36].

Both rotavirus vaccines currently marketed, Rotarix® and RotaTeq®, have demonstrated very good safety and efficacy in large clinical trials, providing protection against homotypic and heterotypic rotavirus strains [12–14, 37]. However, the present study has revealed symptomatic rotavirus infections among fully vaccinated children.

Rotavirus vaccine coverage in our geographical area has been estimated at 42% [23]. A minimum single dose can be viewed as affording protection, because a previous study in our Autonomous Region have shown high protection with partial vaccination [23]. Nevertheless, 7 out of our patients had only received a single vaccine dose, 6 with RotaTeq and 1 with Rotarix. This would imply that partial vaccination does not protect adequately. Higher levels of vaccination among children under 3 years of age was observed; however, it was precisely this age group that experienced the greatest number of infections. The ratio of vaccinated children among rotavirus-infected patients (30.1%) was lower than in the control group (56%) (OR: 2.96, CI95%: 1.51–5.78, *p* = 0.003). It has been claimed that high percentages of rotavirus detection by immunochromatographic method in vaccinated children, could be due to false positives, especially when the test is performed outside rotavirus seasons [38]. However, all the positive results reported in this study were confirmed by RT-PCR, and the infecting rotavirus strains were characterized. It has been reported that vaccinated children may shed vaccine strains in stool samples up to 13 days post vaccination [39]. However, infecting rotavirus genotypes in the children reported in this study are not present in the composition of the vaccines, except G1P[8], or the time frame between last dose and time of infection exceeded potential faecal shedding of vaccine strains in immunocompetent patients [40]. Although none of other investigated enteropathogenic bacteria or viruses were detected in our patients by conventional methods, we cannot completely exclude that more sensitive molecular methods could have detected some coinfection.

Current rotavirus vaccines were designed according to the circulating strains in the 1980s, when G1, G2, G3, G4, P[4] and P[8] were the most prevalent genotypes and G1P[8] was the dominant rotavirus in most Western countries [41, 42]. However, circulating rotaviruses are currently different, having diversified thanks to their evolutionary mechanisms and to genotype fluctuations [43]. In a study conducted in Belgium, significant antigenic differences were demonstrated between the G1 and P[8] components present in vaccine strains and in circulating G1P[8] strains [44]. In addition, a study carried out in Nashville (USA) indicates that the antigenic differences observed between circulating rotavirus and vaccine strains are greater in G9P[8] and G12P[8] than in other genotypes [45]. This fact could reduce selective pressure from neutralizing antibodies in these genotypes, which would explain their global epidemiological increase. Furthermore, the effects of widespread vaccination on rotavirus evolution and the level of cross-protection provided by current vaccines against antigenically distinct rotaviruses remain areas of active investigation [45]. In this study, G9P[8], G12P[8] and G1P[8] were the most frequently detected genotypes in vaccinated children, as well as in the unvaccinated population. Interestingly, a marked increase of rotavirus G12P[8] in the hospital setting was reported in Nicaragua, associated with a nationwide predominance of this genotype in a population with high pentavalent vaccine coverage [46]. It could be considered that some factors might cause a selection or rotavirus strains circulating in the area, and perhaps vaccination could be one of them. In addition, several host factors have been suggested to impact on the protection induced by rotavirus vaccines in children, like breastfeeding, the presence or absence of immune and non-immune components in breast milk and the intestinal bacterial microbiota [47, 48]. Recent studies show that histo-blood group antigens, complex carbohydrates located on the surface of mucosa epithelial cells, play a role in human rotavirus infections [49, 50]. Moreover, the use of rotavirus vaccines may give rise to unexpected consequences, as it has been found that circulating atypical rotavirus strains can originate via reassortment between human G1P[8] and RotaTeq vaccine strains [51]. Pitzer et al. performed a mathematical model to understand the potential impact of vaccination on genotype distributions which suggested that vaccination may influence the selection of the different circulating genotypes [52]. Another study data to corroborate this assertion was inconclusive [43]. Nevertheless, despite some antigenic changes in circulating genotypes, the vaccine showed dramatic effect and reduction in rotavirus diarrhea [52]. In all likelihood, the selection of the circulating rotavirus genotypes that we have observed in this work is more complex, perhaps owing to a set of circumstances that encompass natural evolution, vaccination and other unknown factors. The effects of vaccination on the circulating rotavirus strains are probably more complex than merely serotype selection. Nevertheless, additional monitoring and directed studies will help to clarify the specific roles of vaccines in rotavirus selection and evolution.

## Conclusions

Rotavirus vaccine failures may cause loss of confidence in the vaccine and lower the estimates of vaccine effectiveness. Although the impact of rotavirus vaccines on various commonly circulating strains has been well documented, with a drastic reduction in morbidity and mortality worldwide, rotavirus infections can sometimes occur, especially in populations with low and moderate vaccination coverage. Further studies are needed to explain possible vaccine failures and to clarify the specific role of vaccines in rotavirus selection and evolution. Despite rotavirus vaccination, paediatricians should consider rotavirus as a suspected agent in cases of acute gastroenteritis.

## Data Availability

The datasets used and/or analysed during the current study are available from the corresponding author on reasonable request.

## Abbreviations

ELISA: Enzyme-linked immunosorbent assay
PBS: Phosphate buffered saline
RNA: Ribonucleic acid
RT-PCR: Reverse transcriptase - polymerase chain reaction
